# Estimating the evolutionary rates in mosasauroids and plesiosaurs: discussion of niche occupation in Late Cretaceous seas

**DOI:** 10.7717/peerj.8941

**Published:** 2020-04-13

**Authors:** Daniel Madzia, Andrea Cau

**Affiliations:** 1 Department of Evolutionary Paleobiology, Institute of Paleobiology, Polish Academy of Sciences, Warsaw, Poland; 2 Independent, Parma, Italy

**Keywords:** Mosasauroidea, Plesiosauria, Late cretaceous, Bayesian tip-dating, Rates ofmorphological evolution, Niche occupation

## Abstract

Observations of temporal overlap of niche occupation among Late Cretaceous marine amniotes suggest that the rise and diversification of mosasauroid squamates might have been influenced by competition with or disappearance of some plesiosaur taxa. We discuss that hypothesis through comparisons of the rates of morphological evolution of mosasauroids throughout their evolutionary history with those inferred for contemporary plesiosaur clades. We used expanded versions of two species-level phylogenetic datasets of both these groups, updated them with stratigraphic information, and analyzed using the Bayesian inference to estimate the rates of divergence for each clade. The oscillations in evolutionary rates of the mosasauroid and plesiosaur lineages that overlapped in time and space were then used as a baseline for discussion and comparisons of traits that can affect the shape of the niche structures of aquatic amniotes, such as tooth morphologies, body size, swimming abilities, metabolism, and reproduction. Only two groups of plesiosaurs are considered to be possible niche competitors of mosasauroids: the brachauchenine pliosaurids and the polycotylid leptocleidians. However, direct evidence for interactions between mosasauroids and plesiosaurs is scarce and limited only to large mosasauroids as the predators/scavengers and polycotylids as their prey. The first mosasauroids differed from contemporary plesiosaurs in certain aspects of all discussed traits and no evidence suggests that early representatives of Mosasauroidea diversified after competitions with plesiosaurs. Nevertheless, some mosasauroids, such as tylosaurines, might have seized the opportunity and occupied the niche previously inhabited by brachauchenines, around or immediately after they became extinct, and by polycotylids that decreased their phylogenetic diversity and disparity around the time the large-sized tylosaurines started to flourish.

## Introduction

Marine amniotes underwent changes in the relative contribution of particular clades to overall functional disparity near the Early-Late Cretaceous transition (Stubbs & Benton, 2016). The contribution of plesiosaurs visibly decreased while that of mosasauroids was rising (Stubbs & Benton, 2016: Fig. 3), suggesting that in addition to the extinction of ichthyosaurs and significant reorganization of marine environments, competition between plesiosaurs and mosasauroids might have played a role. Brief discussion of such possibility has previously been provided by Schumacher (2011) following discovery of a russellosaurinan mosasauroid co-occurring with the last brachauchenine pliosaurids.

Origins, diversification, and decline of particular clades of aquatic Mesozoic amniotes are commonly linked to climatic changes and environmental volatility (e.g.,  Benson & Druckenmiller, 2014; Fischer et al., 2016; Tennant, Mannion & Upchurch, 2016). The same applies for mosasauroid squamates whose early evolution in the early Late Cretaceous was hypothesized to have been primarily driven by high marine productivity associated with warm climate and high sea stands (Polcyn et al., 2014). The factors influencing large-scale biotic events, such as turnovers, however, are often tricky to grasp in full, especially if they involve clade-specific interactions, such as competition, that depend on many biological factors (see general studies by Benton, 1983; Benton, 1987; Benton, 1991; and, for example McGowan & Dyke, 2007; Butler et al., 2009; Benson et al., 2014 for detailed assessments of pterosaur-bird competitiveness hypothesis).

This study is aimed to discuss the evolutionary history of mosasauroids, a clade of aquatic squamates known exclusively from the Upper Cretaceous strata (e.g.,  Polcyn et al., 2014; Simões et al., 2017; Madzia & Cau, 2017), through the inference of their rates of morphological evolution. Specifically, the evolutionary rates and traits of mosasauroids are compared to those of plesiosaurs, a successful clade of aquatic amniotes that originated in the Late Triassic and disappeared at the end of the Cretaceous (e.g.,  Ketchum & Benson, 2010; Benson & Druckenmiller, 2014; Wintrich et al., 2017).

Increased dynamics of phenotypic evolution in adaptive radiations is commonly linked with interactions of sympatric species that lead to the phenomenon Darwin (1859) called ‘divergence of character’ (now termed ‘character displacement’; Brown & Wilson, 1956; see also Pfennig & Pfennig, 2010), which maintains that differences in traits of species with similar phenotypes appear to minimize competitive interactions between their populations. This is a ‘small-scale’ process that is well-documented on closely related species (e.g.,  Sætre et al., 1997; Grant & Grant, 2010). On a larger, macroevolutionary scale, however, the contribution of competitive interactions still remains somewhat unclear (e.g.,  Tobias et al., 2014) and contrasting with respect to particular types of traits, such as those associated with resource-use and those involved in social interactions (e.g.,  Drury et al., 2018). In wholly extinct distantly-related clades with some similar traits and comparable ecologies that at least partially overlapped in time and space, such as mosasauroids and plesiosaurs, the role of competition is particularly difficult to infer. Any assessments must be based on data obtained from a highly incomplete fossil record. We assume that if larger-scale competitive interactions between mosasauroids and plesiosaurs took place at a certain time, signals of these interactions might be noticeable in the evolutionary rates of the competing lineages. No connection between evolutionary rates and competitive interactions has ever been tested and higher/lower rates of divergence in these clades may naturally have other causes as well (e.g., environmental). Nevertheless, estimations of the evolutionary rates of the mosasauroids and plesiosaurs that co-occurred and shared a number of traits suggesting that they occupied similar or the same niches, and discussion of traits that are tightly related to niche occupation, could initiate further clade- or trait-specific studies that will elucidate the patterns of niche occupation in Late Cretaceous seas.

In order to assess the rates of morphological evolution among mosasauroids and among different plesiosaur clades during the mid- to Late Cretaceous, we used two species-level phylogenetic datasets, each modified to reflect the current knowledge regarding the morphological traits within both these groups, updated them with stratigraphic information, and analyzed using the Bayesian inference to reconstruct the tree topologies for both clades and times and rates of divergence for their particular branches.

Following the inferred results and comparisons of traits related to niche occupation, possible competitive interactions of mosasauroids and plesiosaurs are discussed within the criteria of these three hypotheses:

(1) First mosasauroids diversified following competition with plesiosaurs.
(2) At least some mosasauroids competed with contemporary plesiosaurs or seized the opportunity and occupied their niches when they were in demise or became extinct.
(3) The fates of plesiosaurs and mosasauroids were independent of each other (no suggested competitive interactions between mosasauroids and plesiosaurs).

## Methods

### Bayesian inference

Both mosasauroid and plesiosaur phylogenetic datasets were analyzed using the protocol discussed in Madzia & Cau (2017), integrating the morphological data matrices with absolute ages of the least inclusive stratigraphic range including each terminal unit. The Sampled Ancestor Fossilized Birth Death Skyline Model (SAFBD) of Gavryushkina et al. (2014) and Gavryushkina et al. (2017) implemented in BEAST 2.4.4. (Drummond et al., 2012; Bouckaert et al., 2014) was used as tree model. Since the character matrices did not include autapomorphies of the sampled taxa, the Lewis’s (2001) model was conditioned to variable characters only using the implementation included in BEAST 2.4.4. Stratigraphic information for the mosasauroid and plesiosaur taxa was taken from the literature (Polcyn et al., 2014; Fischer et al., 2017; respectively), and converted to geochronological ages. Stratigraphic data and age constraints for each terminal were obtained from the Paleobiology Database (http://paleobiodb.org/), checked against the International Chronostratigraphic Chart (v2019/05; http://stratigraphy.org/), and included as uniform prior for tip-dating (Supplemental Information I).

The impact of using (or omitting) age priors incorporating stratigraphic uncertainty in tip-dating has only recently been addressed (Barido-Sottani et al., 2019; Cau, 2019). Note that in their Bayesian analysis of Mosasauroidea, Madzia & Cau (2017) used a punctiform age prior for each terminal taxon (i.e., the mean value of the shortest age range encompassing the stratigraphic uncertainty), thus they did not incorporate age uncertainty in tree reconstruction. Such a strategy may arbitrarily set the age of several taxa sharing the same stratigraphic uncertainty to an identical value, thus enforcing a strictly cladogenetic pattern for their relationships even under the SAFBD model, and biasing tree reconstruction favoring longer ghost lineages. Furthermore, punctiform tip-dating priors may lead to inflated divergence rates for taxonomic units scored from multiple non-contemporary specimens (see Cau, 2019).

The following protocol was used for both mosasauroid and plesiosaur datasets. Each BEAST analysis involved 3 replicate runs (with different random starting trees and random number seeds). Each of the 3 replicate runs involved 10 million steps with sampling every 1,000 generations, with a burnin of 4 million steps. This protocol is similar to the one followed by Simões et al. (2017) but used an additional independent run for each analysis (i.e., three instead of two) and set a more conservative burnin (40% instead of 25%). We used Tracer 1.5 (Rambaut & Drummond, 2009) to determine whether the runs reached stationary phase, and to assess convergence of the independent runs. The post-burnin parameter and tree samples were retained for the analysis and concatenated using LogCombiner in the BEAST package. Estimates (mean and 95% highest posterior density) for all numerical parameters were generated using Tracer 1.5 (Rambaut & Drummond, 2009). We used the MCCT to reconstruct the cladogenetic events (median age) and to infer the divergence rate (the amount of morphological change per branch relative to the whole topology) for both clades. Note that the absolute rate values are inversely related to the sample size (i.e., rate value in a branch is proportional to the probability of sampling each state transition of the clade history in that branch); thus, direct comparisons between the mosasauroid and plesiosaur rate values is meaningless. Given the rate distribution inferred along the MCCT in the two clades, we here define ‘high rates’ all those values equal or higher than the value at the 75 percentile in each rate distribution.

The mosasauroid matrix was acquired from Simões et al. (2017), which is a recent version of the dataset first introduced in Bell’s (1993) PhD thesis and published by Bell (1997). The recent version of Simões et al. (2017) was further modified with respect to some taxon names (as in Madzia & Cau, 2017). In sum, the dataset consists of 44 operational taxonomic units (OTUs) analyzed using 125 characters (see Supplemental Information II for BEAST file, Supplemental Information III for NEXUS file, and Supplemental Information IV for character list).

The phylogenetic assessment of Plesiosauria was performed using a modified version of the dataset first assembled by Benson & Druckenmiller (2014). We first took a recent version of that dataset, published by Madzia, Sachs & Lindgren (2019), and updated it based on personal observations and recently published literature, to include representatives of distinctive plesiosaur clades. The changes include: modifications to the scores of *Brancasaurus brancai* and ‘*Gronausaurus wegneri*’ as in Sachs, Hornung & Kear (2016); addition of *Lagenanectes richterae* from Sachs, Hornung & Kear (2017); addition of *Nakonanectes bradti*, *Albertonectes vanderveldei*, *Aristonectes quiriquinensis*, *Elasmosaurus platyurus*, ‘*Hydralmosaurus serpentinus*’, *Mauisaurus haasti*, ‘*Libonectes*’ *atlasense*, *Terminonatator ponteixensis*, *Tuarangisaurus keyesi*, *Zarafasaura oceanis*, *Kawanectes lafquenianum*, and *Vegasaurus molyi* from Serratos, Druckenmiller & Benson (2017); addition of *Neusticosaurus pusillus* and *Nothosaurus marchicus*, and modifications to the scores of *Yunguisaurus liae* and *Pistosaurus* OTUs as in Wintrich et al. (2017); addition of *Acostasaurus pavachoquensis*, ‘*Kronosaurus*’ *boyacensis*, and *Sachicasaurus vitae* from Páramo-Fonseca, Benavides-Cabra & Gutiérrez (2018), with amended scores for *A. pavachoquensis* and *S. vitae* as in Páramo-Fonseca, Benavides-Cabra & Gutiérrez (2019); modifications to the character scores of *Thililua longicollis* and addition of *Eopolycotylus rankini*, *Manemergus anguirostris*, *Dolichorhynchops tropicensis*, *Georgiasaurus penzensis*, *Dolichorhynchops* sp. (specimen ROM 29010), *Dolichorhynchops herschelensis*, *Sulcusuchus erraini*, and *Mauriciosaurus fernandezi* following Fischer et al. (2018), with amended scores for *Trinacromerum bentonianum*, *Dolichorhynchops osborni*, *Dolichorhynchops bonneri*, *Mauriciosaurus fernandezi*, and *Polycotylus latipinnis* as in Morgan & O’Keefe (2019); addition of *Styxosaurus snowii* from Sachs, Lindgren & Kear (2018); and modifications to the scores of *Kronosaurus queenslandicus* and *Stenorhynchosaurus munozi* following Holland (2018) and Páramo-Fonseca, Benavides-Cabra & Gutiérrez (2019).

It is essential to note that although the elasmosaurid phylogenetic relationships were a subject of several recent papers (e.g.,  Otero, 2016; Sachs, Hornung & Kear, 2016; O’Gorman et al., 2017; Sachs & Kear, 2017; Serratos, Druckenmiller & Benson, 2017; Sachs, Lindgren & Kear, 2018; O’Gorman et al., 2019), interpretations of morphologies observed in some elasmosaurid specimens differ between these studies. See, for example, conflicting scores for ‘*Libonectes*’ *atlasense* in Sachs & Kear (2017) and Serratos, Druckenmiller & Benson (2017), and for *Styxosaurus snowii* in Serratos, Druckenmiller & Benson (2017) and Sachs, Lindgren & Kear (2018). We have not studied these taxa in person; as such, we had to choose between scores provided in other publications. We decided to adopt those scores that derive from more recent studies in which the taxa were assessed based on direct observations. For that reason, ‘*Libonectes*’ *atlasense* is here scored as in Serratos, Druckenmiller & Benson (2017) and *Styxosaurus snowii* as in Sachs, Lindgren & Kear (2018). Naturally, such differences in interpretations of character states might have an impact on inferred evolutionary rates. Nevertheless, our decisions should not have any impact on the findings of the present study as Elasmosauridae is of marginal importance here.

Finally, we have also modified several character states of *Anguanax zignoi* based on personal observations of the type specimen (3: 0→?; 4: [12]→?; 121: 0→?; 137: 0→?; 150: 1→?; 207: [01]→?; 270: 1→0) and re-scored *Megacephalosaurus eulerti* for character 27 (1→0). This score has been already advocated by Madzia, Sachs & Lindgren (2019: p. 1208) but the character was erroneously scored as ‘1’ rather than ‘0’ in that study.

Additionally, we have modified several character definitions in the character list of Benson & Druckenmiller (2014):

*Character 25.* The character description was changed from “Maxilla, posterior extent of maxillary tooth row” to “Maxilla and dentary, posterior extent of maxillary tooth row”; after Serratos, Druckenmiller & Benson (2017).

*Character 138.* As noted by Madzia, Sachs & Lindgren (2019), the current state definitions for character 138 are problematic because they do not cover all plesiosaurs. In the original character list of Benson & Druckenmiller (2014), state ‘0’ was defined as codable for taxa with 12–17 maxillary teeth, state ‘1’ for taxa with 20–25 maxillary teeth, and state ‘2’ for taxa with more than 28 maxillary teeth. However, the brachauchenine pliosaurid *Megacephalosaurus eulerti* was shown to possess 18 teeth in the right and 19 in the left maxilla, thus falling between states ‘0’ and ‘1’. Two options were considered for *M. eulerti*: to score it as ‘0’, extending the state to cover taxa with 12–19 maxillary teeth, and as ‘1’, extending the state to cover taxa with 18–25 teeth in their maxillae. Madzia, Sachs & Lindgren (2019) used both these options and explored the effects of such settings. Considering that the last brachauchenines have reduced numbers of teeth in their jaws (Madzia, Sachs & Lindgren, 2019), scoring these taxa in the same way as their older relatives (that fall near the upper boundary of state ‘1’) might hinder the inference of some potential phylogenetic signal. Therefore, in this study, state ‘0’ covers taxa with 12–19 maxillary teeth, state ‘1’ covers taxa with 20–27 maxillary teeth (note that the upper boundary was extended to eliminate the gap between states ‘1’ and ‘2’), and state ‘2’ covers taxa with at least 28 maxillary teeth.

*Character 139*. State ‘2’ (“intermediate between states 0 and 1, with a flattened labial surface, but this surface [is] not substantially expanded anteroposteriorly [= subtrihedral]”) was added after Benson et al. (2013). Even though Benson et al. (2013) described the state ‘2’ as “intermediate”, the morphological transition from “round or sub-rounded” (‘0’) to “sub-triangular [= trihedral]” (‘1’) does not need to have the appearance of ‘2’. Later, Serratos, Druckenmiller & Benson (2017) used the dataset of Benson & Druckenmiller (2014) to infer the interrelationships of elasmosaurids and modified character 139 to include another new state (‘2’): “suboval”. However, in their data matrix, this state was scored as ‘3’. In this study, the state ‘2’ is equivalent to state ‘2’ of Benson et al. (2013), and state ‘3’ follows the new “suboval” state introduced by Serratos, Druckenmiller & Benson (2017). Nevertheless, the perception of what is “sub-rounded” (‘0’) and what “suboval” (3) may be partially dependent on subjective criteria. As such, future larger-scale phylogenetic studies of Plesiosauria should probably quantify the difference (for example, using the ‘width-to-length ratio’ [WLR] of Madzia (2016) or similarly defined parameter). Due to the lack of apparent transitional nature of particular character states, this character should stay unordered in parsimony analyses.

*Character 153*. State ‘3’ (“even longer, corresponding to the ‘can’ shaped morphology of Otero et al. (2016a) or ‘elongate’ morphology of O’Keefe & Hiller (2006)”) was added after Serratos, Druckenmiller & Benson (2017). We did not modify the state definition though, again, its quantification would enable to maintain an unambiguous application.

*Character 248*. The character description was changed from “Propodials, angle between long axes of epipodial facets in dorsal view” to “Humerus, angle between long axes of epipodial facets in dorsal view”; after Serratos, Druckenmiller & Benson (2017).

The full dataset consists of 125 OTUs analyzed using 270 characters (see Supplemental Information V for BEAST file, Supplemental information VI for NEXUS file, and Supplemental information VII for character list). This is the largest dataset used in phylogenetic assessment of Plesiosauria published to date.

### Reconstruction of geographic origin

**Figure 1:**
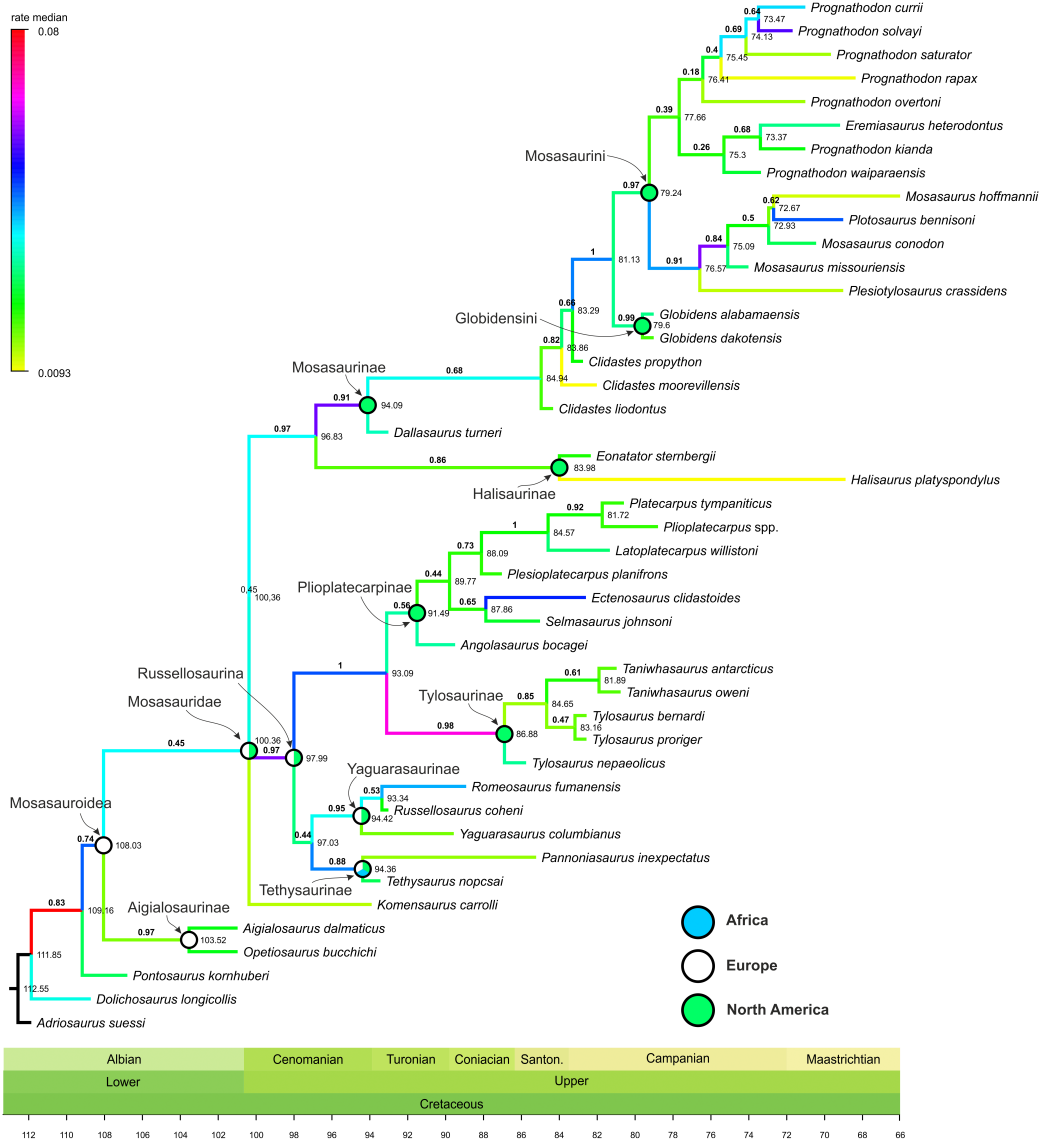
Maximum Clade Credibility Tree (MCCT) of Mosasauroidea. MCCT of Mosasauroidea, rooted on *Adriosaurus*. Branches colored according to median rate of divergence. Time scale in My. Values above branches (bold) indicate the posterior probability of the clade; values at nodes indicate median age of divergence of node (Ma). Circles on nodes indicate areas of origin reconstructed for major mosasauroid clades.

**Figure 2:**
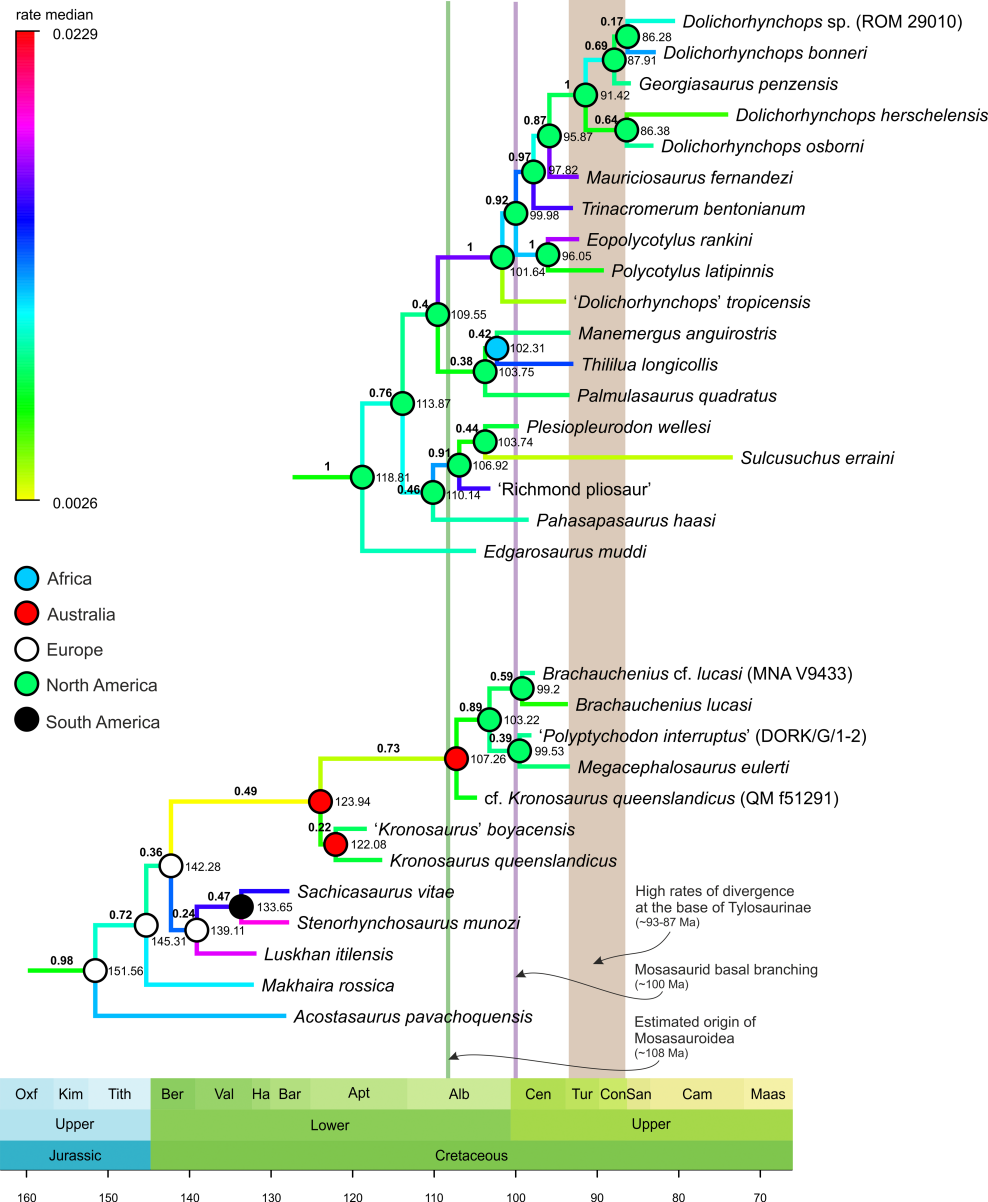
Selected segments of the Maximum Clade Credibility Tree (MCCT) of Plesiosauria. The topologies and rates of divergence for brachauchenine pliosaurids and polycotylid leptocleidians, with some major events in the evolutionary history of Mosasauroidea. Branches colored according to median rate of divergence. Time scale in My. Values above branches (bold) indicate the posterior probability of the clade; values at nodes indicate median age of divergence of node (Ma). Circles on nodes indicate reconstructed areas of origin.

A detailed assessment of the historical biogeography of mosasauroids and plesiosaurs is beyond the scope of our study; however, for the sake of discussion related to possible competitive interactions of mosasauroids and plesiosaurs, we provide a brief insight into possible geographic origins for mosasauroid and plesiosaur clades. We took the MCCTs resulting from the Bayesian analyses of both datasets, loaded them to Mesquite 3.61 (Maddison & Maddison, 2019) and created a new character matrix with a single new character scored for each OTU—the continent of discovery. Such demarcation only very roughly corresponds to the real ancient biogeographic settings (such as Cretaceous epicontinental seas); however, for the purposes of our study such highly simplified approach seems to be sufficient as the ancestral areas in the time interval that is of special interest for us (e.g., the Western Interior Seaway [covered under ‘North America’] in the Turonian; see Figs. 1 and 2) do not seem to comprise water bodies with geographically isolated populations.

The geographic origins for particular clades were reconstructed using the ‘Trace Character History’ option and with ‘Parsimony Ancestral States’ as the ancestral state reconstruction method. The resulting ancestral areas were mapped on Figs. 1 and 2.

## Results

### Rates of morphological evolution

The rate of evolutionary divergence inferred among the branches of the mosasauroid MCCT (both internodes and terminal leaves; Fig. 1) ranges between 0.0093 and 0.0800 changes per branch (median value = 0.0289, 25–75 percentile range: 0.0195–0.0367). During their evolutionary history mosasauroids experienced intervals of elevated rates of divergence. High rates are especially apparent at the origin of Russellosaurina (∼100–98 Mya; rate = 0.0588), the clade consisting of plioplatecarpines and tylosaurines (∼98–93 Mya; rate = 0.0472), the base of Tylosaurinae (∼93–87 Mya, rate = 0.0674), and near the base of Mosasaurinae (∼97–94 Mya; rate = 0.0577). An increase in rates of morphological evolution is also apparent at the base of the node comprising *Mosasaurus* and *Plotosaurus* (∼77–75 Mya; rate = 0.0588).

Only three plesiosaur lineages might have affected mosasauroid evolution or been affected by interactions with them. These include the polycotylid leptocleidians, elasmosaurids, and brachauchenine pliosaurids. However, with their elongated necks and long and pointed teeth, elasmosaurids were substantially distinct from all known members of Mosasauroidea, thus occupying dissimilar niches (e.g., Massare, 1987). As such, additional comparisons will be limited only to the brachauchenine and polycotylid plesiosaurs (see Fig. 2; due to the large size of the MCCT of Plesiosauria, the full tree is provided as the Supplemental Information VIII).

The origin of the least inclusive clade formed by Russellosaurina and Mosasaurinae (+ Halisaurinae) occurred ∼100 Mya, and the splitting of the clade uniting plioplatecarpines and tylosaurines ∼93 Mya; thus covering the last few millions of years of the brachauchenine evolution. In turn, during the splitting of the smallest clade comprising globidensins and mosasaurins (∼81 Mya) and the origin of the *Mosasaurus* node (∼75 Mya), pliosaurids were probably already extinct and polycotylids rare (e.g., Sato, 2005; Salgado, Parras & Gasparini, 2007; O’Gorman & Gasparini, 2013; Novas et al., 2015; Fischer et al., 2018; Morgan & O’Keefe, 2019).

The last increase in the rates of morphological evolution within Pliosauridae occurred at the base of the clade formed by *Luskhan itilensis*, *Stenorhynchosaurus munozi*, and *Sachicasaurus vitae* (∼142–139 Mya) and within that clade. Last brachauchenines of the early Late Cretaceous (Cenomanian-Turonian) have not experienced significant oscillations in their evolutionary rates, indicating potential evolutionary conservatism among the last pliosaurids.

In contrast, polycotylids experienced elevated rates in the ‘middle’ Cretaceous members of the clade.

(See Supplemental Information IX for resulting ‘log’ files from the analyses of both datasets).

### Bayesian analysis of plesiosaur phylogenetic relationships

The phylogenetic relationships of Mosasauroidea inferred by means of Bayesian analysis of the dataset presented herein have already been thoroughly discussed in Simões et al. (2017) and Madzia & Cau (2017). Owing to the fact that the overall tree topology of the mosasauroid MCCT is the same as in Madzia & Cau (2017), with minor differences in the mosasauroid outgroup and in the ‘*Prognathodon*’ and ‘*Mosasaurus*’ grouping, detailed discussion of the results is provided only for the plesiosaur tree (see Supplemental Information VIII for the full tree).

Inference of plesiosaur interrelationships through Bayesian analysis, including estimates of rates of divergence for some clades, has previously been published as well (Cau & Fanti, 2016); however, the matrix in that study was significantly reduced to include only 39 OTUs. Pliosaurids have been represented as in Benson et al. (2013), the study that Cau & Fanti (2016) took the dataset from, with the only addition being their newly-established pliosaurid taxon *Anguanax zignoi*. The representatives of other major clades (rhomaleosaurids and plesiosauroids) were mostly excluded. Anyway, the original dataset has been substantially expanded and modified since 2016 (see some of the recent additions and modifications in ‘Methods’). Furthermore, there are two substantial methodological differences between the Bayesian inference of Cau & Fanti (2016) and the one performed in the present study:

(1) In the present study, the age uncertainty of each taxon is incorporated in the analysis: age prior of all fossil taxa is defined as a uniform range including the absolute age limits of the shortest stratigraphic range unambiguously including any taxon. This approach differs from that followed in Cau & Fanti (2016), who used for each tip a fixed age prior defined arbitrarily by the mean value of the stratigraphic uncertainty of each taxon.
(2) The tree model used here discriminates between anagenetic and cladogenetic patterns of evolution; therefore, it may test whether some of the taxonomic units that are included actually form anagenetic sequences. The analysis in Cau & Fanti (2016) was run on a previous version of BEAST which did not implement the Sampled Ancestor Fossilized Birth Death Skyline Model (Gavryushkina et al., 2014), and thus was *a priori* constrained to reconstruct exclusively cladogenetic frameworks.

As discussed by Cau (2019), the results of the analysis of Cau & Fanti (2016) may thus be methodologically biased in potentially inflating the extent of the inferred ghost lineages and also in pre-dating the ages of the divergence events.

The present study includes the first Bayesian analysis of the full dataset, with most representatives of all major plesiosaur clades, and including the aforementioned modifications.

The overall tree topology broadly agrees with those reconstructed through more recent parsimony analyses (see, e.g., Ketchum & Benson, 2010; Benson et al., 2013; Benson & Druckenmiller, 2014; Fischer et al., 2015; Cau & Fanti, 2016; Otero, 2016; Sachs, Hornung & Kear, 2016; Fischer et al., 2017; O’Gorman et al., 2017; Sachs, Hornung & Kear, 2017; Serratos, Druckenmiller & Benson, 2017; Fischer et al., 2018; O’Gorman, Gasparini & Spalletti, 2018; Páramo-Fonseca, Benavides-Cabra & Gutiérrez, 2018; Sachs, Lindgren & Kear, 2018; Madzia, Sachs & Lindgren, 2019; Morgan & O’Keefe, 2019). Plesiosauria (posterior probability [*pp* ] = 1; node origin estimated at ∼241 Mya) basally branches into Rhomaleosauridae (*pp* = 0.96; ∼215 Mya) and Neoplesiosauria (*pp* = 0.88; ∼215 Mya), consisting of Pliosauridae (*pp* = 1; ∼206 Mya) and Plesiosauroidea (*pp* = 0.89; ∼210 Mya). Within the pliosaurid branch, two nodes have been named—Thalassophonea (*pp* = 0.59; ∼174 Mya) and Brachaucheninae (*pp* = 0.98; ∼152 Mya). Interestingly, contrary to recent studies assessing the phylogenetic relationships of pliosaurid plesiosaurs by means of parsimony analyses (e.g., Fischer et al., 2015; Fischer et al., 2017; O’Gorman, Gasparini & Spalletti, 2018; Páramo-Fonseca, Benavides-Cabra & Gutiérrez, 2018; Madzia, Sachs & Lindgren, 2019), the monophyly of *Pliosaurus* may be considered supported (*pp* = 0.83). All ‘major’ plesiosauroid sub-clades appear to be very well supported by our Bayesian analyses (with possible exception to the basal branching of Elasmosauridae; see below). The clade Cryptoclidia (*pp* = 1; ∼180 Mya) consists of Cryptoclididae (*pp* = 1; ∼176 Mya) and Xenopsaria (*pp* = 1; ∼158 Mya), which, then, comprises the clade Leptocleidia (*pp* = 1; ∼145 Mya)—including Leptocleididae (*pp* = 0.9; ∼140 Mya) and Polycotylidae (*pp* = 1; ∼119 Mya)—and its closest relatives (*Brancasaurus* and ‘*Gronausaurus*’), and the clade Elasmosauridae (*pp* = 0.44; ∼144 Mya). However, the low *pp* value for the base of Elasmosauridae might be due to the problematic placement of *Lagenanectes richterae* which might be a non-elasmosaurid xenopsarian (D. Madzia, unpublished results). The *pp* value of the more ‘traditional’ elasmosaurid grouping (that is, exclusive of *L. richterae*) is very high (*pp* = 0.99).

## Discussion

### Estimates of evolutionary rates and potential biases

#### The impact of phylogenetic uncertainties on inferences of evolutionary rates

Both mosasauroid and plesiosaur datasets include shortcomings that might have had an impact on the inferences of the rates of morphological evolution within these groups. The dataset used for the assessment of the phylogenetic relationships within mosasauroids has been reviewed and discussed by Madzia & Cau (2017) who recommended that the currently inferred topologies should be seen as tentative pending extensive modifications to the data sampling. Nevertheless, Madzia & Cau (2017) also noted that analyses using multiple phylogenetic methods revealed general congruencies regarding monophyly of major mosasauroid groups (mosasaurines, tylosaurines, plioplatecarpines, halisaurines, tethysaurines, and possibly also yaguarasaurines). Differences involved their mutual relationships which remain largely unsettled. At the same time, elevated rates of morphological evolution have been inferred for well-supported clades with posterior probability (*pp*) values around 0.9 and often higher (Plioplatecarpinae + Tylosaurinae: *pp* = 1; Tylosaurinae: *pp* = 0.98; Mosasaurinae: *pp* = 0.91; Globidensini + Mosasaurini: *pp* = 1; *Mosasaurus*: *pp* = 0.84; see Fig. 1). Given such results, it seems probable that the rates inferred for major or well-supported mosasauroid nodes would stay similar even after the changes to the dataset suggested by Madzia & Cau (2017).

Similarly, there do not seem to be any doubts regarding the composition of advanced brachauchenines and polycotylids, the two groups that are compared here with mosasauroids.

#### Sampling bias

Any inferences of the rates of morphological evolution by means of Bayesian phylogenetics can or should be based only on reasonably complete material. That means, for example, that isolated fragments often have to be omitted from the datasets as the methods cannot handle them or may produce dramatically labile relationships. Still, they might provide important information on the ‘persistence’, diversity, and geographic distribution of particular lineages. For instance, fragmentary material suggests that the brachauchenine lineage might have reached the latest middle Santonian (∼84 Ma), as might be evidenced by an isolated tooth crown originating from the *Micraster coranguinum* Zone (the uppermost lower Coniacian to the uppermost middle Santonian) of the Seaford Chalk Formation, Gravesend, Kent, England (Madzia, 2016). Nevertheless, the material is too limited to serve as an indicator of the lineage ‘vitality’ and competitiveness. Instead, if really belonging to a brachauchenine (and not a robust-toothed polycotylid), it seems to merely represent a relict of the once widespread pliosaurids. In turn, other isolated elements belonging to pliosaurids and polycotylids, such as those studied by Kear et al. (2014), Madzia & Machalski (2017), Sachs et al. (2017), Sachs et al. (2018), Sachs et al. (2020), and Zverkov & Pervushov (2020) might suggest a higher taxic diversity and a wider geographic distribution of the clades during the Albian–Turonian (‘middle’ Cretaceous).

Similarly, the large-scale assessment of pliosaurid teeth by Zverkov et al. (2018), that was based on material representing the vast majority of pliosaurid taxa, including assemblages of isolated teeth, suggested that pliosaurids (1) could have been represented by two lineages in the Cretaceous, instead of one, and that (2) they experienced the highest dental disparity around the Jurassic/Cretaceous boundary interval. Such results were surprising because they were and still are in striking contrast with all studies assessing the phylogenetic relationships of Pliosauridae, that cannot consider numerous latest Jurassic/Early Cretaceous specimens due to their highly fragmentary nature, and that suggest the presence of a single clade of pliosaurids crossing the Jurassic/Cretaceous boundary. Newer studies, such as that of Lukeneder & Zverkov (2020) provide further support for the findings of Zverkov et al. (2018).

### Comparing mosasauroid and plesiosaur traits

#### Tooth crown morphologies and trophic guilds

**Figure 3:**
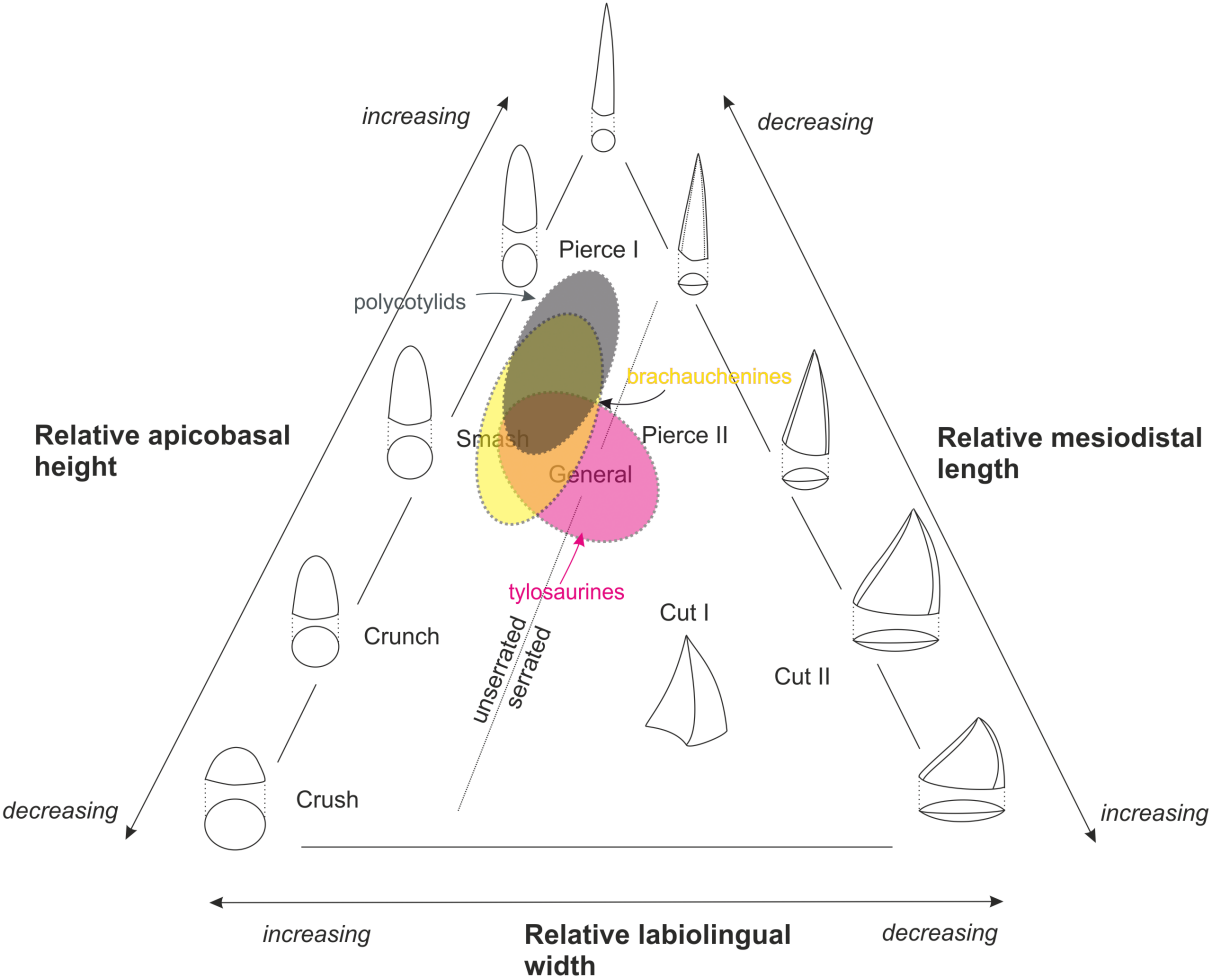
Massare’s (1987) ternary graph, as modified by Hornung & Reich (2015), showing the association of tooth crown morphologies and function. An approximate partial overlap of the tylosaurine dental morphospace with that of brachauchenine pliosaurids and polycotylid leptocleidians.

Driven by “a lack of precision in correlating tooth type and preferred prey” in Mesozoic marine amniotes, Massare (1987) designed seven trophic guilds based on tooth crown morphologies: (I) crush, (II) crunch, (III) smash, (IV) pierce I, (V) pierce II, (VI) general, and (VII) cut (Massare, 1987: 130–131), and provided characteristics of particular crown morphologies used in determining assignment to guilds (Massare, 1987: 132, Table 3). Her classification has been further modified by Hornung & Reich (2015) who divided the ‘cut’ guild into two categories, ‘cut I’ and ‘cut II’, to distinguish between taxa with labiolingually expanded crown with cutting edges (‘cut I’) from those with strongly compressed and blade-shaped teeth (‘cut II’) (see Fig. 3). Even though the guild classification proposed by Massare (1987) has not been accepted universally (Buchy, 2010), recent quantitative studies using teeth of marine amniotes to evaluate feeding ecologies validated such system (e.g., Foffa et al., 2018).

The Late Cretaceous brachauchenines and polycotylids can be categorized relatively easily within that system. With their conical and slightly curved teeth, both these clades belong to the non-carinate/unserrated ‘general’/‘smash’/‘pierce I’ guilds, though polycotylids usually possess less robust teeth leaning further towards the ‘pierce I’ part of the system (Fig. 3). The guild assignment of the mosasauroids is problematic due to the apparent and widespread pseudoheterodonty in some clades. Specifically, the shape of the mosasauroid teeth often differs depending on the positions of the teeth in jaws (see, e.g., LeBlanc, Caldwell & Bardet, 2012; Otero et al., 2016b; Madzia, 2019). For example, the derived mosasaurine mosasaurid *Mosasaurus lemonnieri*, which is known from a number of well-preserved specimens with reasonably complete jaws, shows anterior-to-middle tooth crowns with subcircular/ovoid cross-sections and carinae variably developed either on a short apical segment of the tooth crowns (in IRSNB R 366, 368, 369) or along their entire apicobasal length (serrated only in the somewhat problematic specimen IRSNB R 377). Its posteriorly positioned teeth, in turn, tend to be increasingly labiolingually compressed (Madzia, 2019). Further differences are appearing through the ontogeny as larger (presumably older) individuals appear to show more robust teeth. Such morphological diversity of its tooth crowns makes the taxon occupying a wide field of the ternary graph (from ‘pierce I’/‘pierce II’ through the ‘cut I’/‘cut II’ guilds). In tylosaurines, in turn, the teeth occupy the ‘smash’ to ‘general’/‘pierce II’ field, and mosasaurins show dental morphologies indicative of the ‘crunch’/‘general’ to ‘cut I’/‘cut II’ guilds (Schulp et al., 2006; Ross, 2009; Hornung & Reich, 2015). Therefore, many mosasauroids could likely occupy the same trophic levels of generalists and represent the same trophic guilds as robust-toothed short-necked plesiosaurs.

**Figure 4:**
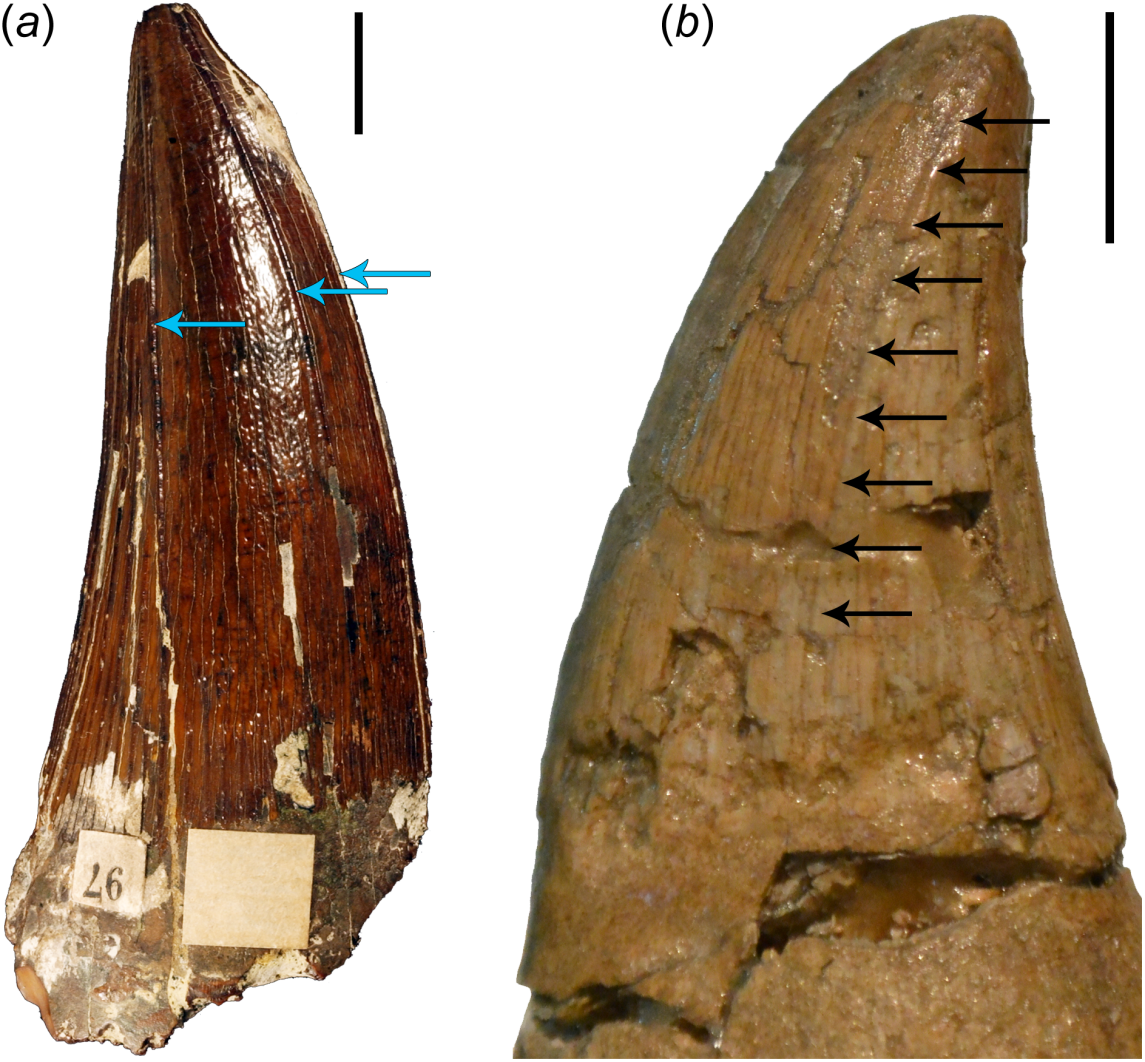
Comparisons of brachauchenine and tylosaurine tooth crowns. (a) Isolated tooth crown (CAMSM B 57378) attributed to the dubious brachauchenine taxon ‘*Polyptychodon interruptus*’ (described and pictured by Madzia, 2016), from the Cambridge Greensand Member of the West Melbury Marly Chalk Formation in labial view; showing the distribution and development of the apicobasal ridges (indicated by blue arrows); and (b) left dentary tooth crown (ld04) of IRSNB R 23 (type of *Tylosaurus bernardi*) in labial view. Arrows on (b) show a labial displacement of the distal carina; resembling the apicobasal ridges in plesiosaurs. Scale bars = 1 cm. Photograph credits: Daniel Madzia.

It is also essential to note here that even though most mosasauroids possess carinate teeth (which distinguishes them from the teeth of the last brachauchenines as well as polycotylids), their distal carinae are often displaced labially (see Fig. 4), especially in the teeth from the anterior to middle section of the jaws. In such cases, the distal carinae resemble (and are often less pronounced than) the apicobasal ridges observable in plesiosaur teeth (see Fig. 4 and the distribution of apicobasal ridges in brachauchenine teeth in Madzia (2016), discussion of outer enamel structural elements in pliosaurid teeth by Zverkov et al. (2018), and assessment of ridge evolution and function in marine amniotes by McCurry et al. (2019)) and certainly does not play the role of a cutting edge (as in labiolingually strongly compressed tooth crowns).

#### Body size evolution

The earliest mosasauroids, such as *Aigialosaurus*, *Carsosaurus*, *Haasiasaurus*, *Komensaurus*, and *Opetiosaurus*, had slim and elongated bodies, with a total length of about 1 to 2 m, which strongly contrasts with the bulky, multitone contemporary plesiosaurs. However, larger-sized mosasauroids appeared relative early in the evolutionary history of the clade, as is documented, for example, by the specimen TMM 43345-1, which represents a large tylosaurine (Bell, Barnes & Polcyn, 2013). The specimen originates from the upper middle Turonian of the Ernst Member (Boquillas Formation) of Texas; thus, tylosaurines evolved larger-sized forms around the time or shortly after brachauchenines became extinct. Nevertheless, members of other mosasauroid clades (plioplatecarpines and mosasaurines) evolved large sizes (>5 m in length) relatively early as well (see, e.g., Polcyn et al., 2014: Appendix A. Supplementary data).

#### Evolution of swimming abilities

Swimming modes and abilities of Mesozoic aquatic vertebrates are tightly connected with their physiology, behavior, and other aspects of their biology and, thus, constitute an important research area (e.g., Massare, 1988; Massare, 1994; Motani, 2002). A number of studies have thoroughly assessed the swimming abilities in plesiosaurs and discussed the differences in the two main plesiosaur ‘body plans’ –the ‘long-’ and ‘short-necked’ forms (e.g., Frey & Riess, 1982; Tarsitano & Riess, 1982; Godfrey, 1984; Halstead, 1989; Nicholls & Russell, 1991; Lingham-Soliar, 2000; O’Keefe, 2001; Carpenter et al., 2010; Liu et al., 2015; Muscutt et al., 2017; Noè, Taylor & Gómez-Pérez, 2017; Troelsen et al., 2019). The same applies to mosasauroids whose swimming abilities and especially their origin have been assessed through detailed studies of various aspects of their anatomy (see, e.g., Lindgren, Jagt & Caldwell, 2007; Lindgren et al., 2010; Lindgren, Polcyn & Young, 2011; Konishi et al., 2012; LeBlanc, Caldwell & Lindgren, 2013; Lindgren, Kaddumi & Polcyn, 2013; Houssaye & Bardet, 2013; Cuthbertson et al., 2015; D’Emic, Smith & Ansley, 2015). The mode of swimming in the two clades is known to have differed greatly. Plesiosaurs have exhibited a limb-based propulsion while mosasauroids employed lateral undulatory locomotion. These differences also apparently reflect the modes of predation in these groups. Owing to their anatomical similarities—large heads, relatively short necks, bulky bodies, and rather short tails—brachauchenines and polycotylids were specialized for maneuverability and pursuit (e.g., O’Keefe, 2001). Mosasauroids, in turn, had long been characterized as being slower-swimming predators adapted for brief ambush pursuits (e.g., Massare, 1988; Massare, 1994; Motani, 2002). Over the last few years, however, the knowledge of the mosasauroid body plan evolution has improved considerably (see, e.g., discussions in Lindgren, Polcyn & Young (2011) and Lindgren, Kaddumi & Polcyn (2013), suggesting that the swimming performance of derived mosasauroids was similar to that of pelagic sharks.

In general, mosasauroids comprised a wide array of taxa; from semi-aquatic forms (e.g., Bell & Polcyn, 2005; Polcyn & Bell, 2005; Dutchak & Caldwell, 2006; Dutchak & Caldwell, 2009; Caldwell & Palci, 2007; Mekarski et al., 2019) to fully aquatic swimmers (see, e.g., Lindgren, Jagt & Caldwell, 2007). Nevertheless, the course and timing of their transition from semi- to fully aquatic morphologies (that is, from ‘plesiopedal-plesiopelvic’ to ‘hydropedal-hydropelvic’ conditions; *sensu* Bell & Polcyn (2005) and Caldwell & Palci (2007), a key aspect when considering potential competitive interactions between mosasauroids and plesiosaurs, is somewhat hindered by conflicting hypotheses of the early evolution of the group. Current phylogenetic assessments of the mosasauroid basal branching are highly dependable on the tree-search strategies used (Simões et al., 2017; Madzia & Cau, 2017). For instance, out of several phylogenetic methods applied, only the parsimony analysis with implied weighting performed by Simões et al. (2017), with the default setting of the concavity parameter (*K* = 3), inferred a single origin of the fully aquatic lifestyle in mosasauroids (with a reversal to the ‘plesiopelvic’ condition in tethysaurines). Madzia & Cau (2017) questioned these findings on the ground of the ongoing debate regarding the meaning of the *K* parameter (O’Reilly et al., 2016; Congreve & Lamsdell, 2016; Goloboff, Torres & Arias, 2018) and also the lack of multiple approaches to the phylogenetic assessment using the implied weighting function (see Goloboff, 1993; Goloboff, 1995; Goloboff et al., 2008; Goloboff, Torres & Arias, 2018). It is also essential to note that the use of the *K*-value that is set as default in TNT (that is, *K* = 3), appears to be too strong and leading to unnatural grouping of OTUs, especially for larger datasets (Goloboff, Torres & Arias, 2018). Thus, higher (though not too high) values should be preferred (see also discussion in Herne et al., 2019: Supplemental text S1: 9–12]).

Considering the results stemming from the most recent parsimony and Bayesian analyses (Simões et al., 2017; Madzia & Cau, 2017; this study), mosasauroids might have evolved the fully aquatic lifestyle more than once. Still, the course of the transition remains a subject for detailed multidisciplinary assessments. For example, despite that the study of Houssaye et al. (2013) was focused on mosasaurine mosasauroids, the authors analyzed the limb-bone osteohistology in a wide variety of taxa, including the basal mosasaurine *Dallasaurus*, a taxon with a ‘plesiopedal’/‘hydropelvic’ morphology that is ‘transitional’ between semi- and fully-aquatic forms, as well as specimens assigned to the fully aquatic (‘hydropedal’/‘hydropelvic’) taxa *Clidastes*, *Globidens*, *Mosasaurus*, *Plotosaurus*, and ‘*Prognathodon*’. The results, when further compared with previous osteohistological studies (e.g., Houssaye & Bardet, 2013), revealed that ‘transitional’ mosasauroids, or at least those representing forms intermediate between basal semi-aquatic mosasauroids and advanced fully-aquatic mosasaurines, exhibited a peculiar inner bone organization characterized by the combination of terrestrial-like and aquatic features that suggested a more gradual adaptation to open marine environments than previously thought. Interestingly, the acquisition of the ‘hydropedal’ and ‘hydropelvic’ conditions, as inferred through our Bayesian analysis, occurred approximately at the time and within the lineages with the highest rates of evolution, resulting in the appearance of good swimmers around the time the brachauchenines experienced low rates of morphological evolution and died out.

#### Thermoregulation and metabolic rates

Oxygen isotope compositions (*δ*^18^O) data obtained from the tooth phosphate of plesiosaurs suggest that they were able to regulate their body temperature independently of the surrounding waters and had high metabolic rates that are required for fast swimming over large distances and predation (Bernard et al., 2010). Plesiosaur metabolic rates have been later independently assessed through the study of their osteohistology (Fleischle, Wintrich & Sander, 2018), which supported the inference of high rates in the clade. In mosasauroids, the available evidence offers slightly ambiguous results. Bernard et al. (2010) proposed that the body temperature of mosasauroids could have been at least partly influenced by ambient conditions; still, they found support for high metabolic rates in mosasauroids. While reassessing the results of Bernard et al. (2010) and Motani (2010) noted that the temperatures provided in Bernard et al. (2010) might be artifacts arising from time-dependent depletion of *δ*^18^O (see also Veizer, Godderis & François, 2000), and argued that mosasauroids might have had a tendency to overheat, proposing that they may have been gigantothermic. Nevertheless, such conclusions appear to be in disagreement with a further stable oxygen isotope study (Harrell, Pérez-Huerta & Suarez, 2016) that characterized mosasauroids as being endotherms rather than gigantotherms. With respect to the mosasauroid metabolic rates, Houssaye et al.’s (2013) osteohistological study showed that their basal metabolic rates were intermediate between those of the extant leatherback turtles (that are homeothermic but not endothermic; e.g., Motani (2010) and Houssaye (2013) and those inferred for plesiosaurs (that are endothermic e.g., Bernard et al., 2010; Motani, 2010; Houssaye, 2013; Fleischle, Wintrich & Sander, 2018; Fleischle et al., 2019).

#### Reproduction and early life history

Available evidence related to the reproductive strategies and early life history in mosasauroids and plesiosaurs is currently limited to a few studies (e.g., Caldwell & Lee, 2001; Kear, 2007; O’Keefe & Chiappe, 2011; Houssaye & Tafforeau, 2012; Houssaye & Bardet, 2013; Field et al., 2015) and reports that have not been published beyond conference abstracts (e.g., Bell et al., 1996; Everhart, 2002; Bell & Sheldon, 2004). A study describing the first gravid plesiosaur, a polycotylid specimen referred to *Polycotylus latipinnis* (O’Keefe & Chiappe, 2011), has initiated comparisons between reproductive strategies of plesiosaurs and other marine amniotes, including mosasauroids. Both clades, mosasauroids and plesiosaurs, have been viviparous though their reproductive strategies differed. In the early (semi-aquatic) mosasauroid *Carsosaurus*, females have been apparently giving birth to at least four progenies (Caldwell & Lee, 2001). Published record does not provide definitive answer regarding the number of embryos in more advanced (larger and fully aquatic) members of Mosasauroidea though preliminary reports suggested that plioplatecarpines were giving birth to multiple progenies as well (Bell & Sheldon, 2004). The gravid plesiosaur specimen, in turn, shows only a single fetus (O’Keefe & Chiappe, 2011). Following comparisons of the traits observed in that specimen to those in the closest extant ecological analogs (odontocete cetaceans) and taxa with some plesiosaur-like reproductive traits (*Egernia* spp.), O’Keefe & Chiappe (2011) suggested that plesiosaurs were *K*-selected and hypothesized that they were social and may have been engaged in parental care. It could be speculated that multiple progenies in mosasauroids, if also present in large-sized forms, might have given these squamates some advantages over plesiosaurs, especially if they were born in open pelagic setting and immediately occupied it (e.g., Houssaye & Tafforeau, 2012; Field et al., 2015). When such things are considered, it is worth noting, however, that the theory of *r*/*K*-selection of MacArthur & Wilson (1967), a paradigm popular as a predictive model for life-history evolution in the late 1960s and 1970s (see also Pianka, 1970), has long been challenged (see, e.g., discussion in Reznick, Bryant & Bashey, 2002). Even if mosasauroids and plesiosaurs differed in both, their reproductive strategies and early life history, the evolutionary meaning of these differences and their impact on the life history of mosasauroids and plesiosaurs, when assessed from the perspective of their niche occupation, remains unknown.

### The record of interactions between mosasauroids and plesiosaurs

It is beyond doubt that sympatric mosasauroids and plesiosaurs interacted at the individual scale. Direct evidence, however, is scarce. Everhart (2004) published on a partial plesiosaur specimen preserved as the stomach contents of an 8.8-meter-long adult of *Tylosaurus proriger*. It was discovered in 1918 in the lower Campanian strata of the Niobrara Formation, near Twin Butte Creek, Logan County, Kansas, and first mentioned by Sternberg (1922) but it has not been properly described until 2004. Everhart (2004) admitted a poor state of preservation of the plesiosaur remains but suggested that they most likely belong to the polycotylid *Dolichorhynchops osborni*. Einarsson et al. (2010), in turn, described a plesiosaur propodial of latest early Campanian age, discovered at the Åsen locality, Kristianstad Basin, Sweden. Though incomplete, the specimen was identified as an indeterminate polycotylid. It possesses a distinctive bite mark that was interpreted by Einarsson et al. (2010) to be caused by a large mosasaurine comparable to *Dollosaurus*. Nevertheless, none of these finds could have been unequivocally inferred as representing either predatory or scavenging behavior. Further finds, of interest with respect to early mosasauroid diversification patterns, include mosasauroid and brachauchenine specimens that overlap in time and space (see specimens discussed in Martin & Stewart, 1977; Polcyn et al., 2008; Schumacher, 2011; Kear et al., 2014; Everhart, 2016). These discoveries, however, do not show any evidence of direct interactions between the members of the two clades.

### Concluding remarks

We provide the first estimates of the evolutionary rates for mosasauroid and plesiosaur clades and use the results as a baseline for discussion and comparisons of traits that might have had some impact on the shape of the niche structures in Late Cretaceous seas. Owing to the known stratigraphic distribution of the mosasauroid and plesiosaur lineages, only three plesiosaur clades might have competed with mosasauroids; the elasmosaurids, brachauchenine pliosaurids, and polycotylid leptocleidians. However, considering the overall body plans of the taxa belonging to these groups, and their tooth crown morphologies, which are key indicators of dietary niche partitioning (e.g., Massare, 1987; Schulp et al., 2013; Hornung & Reich, 2015; Foffa et al., 2018), we suggest that only brachauchenines and polycotylids might have been possible niche competitors of mosasauroids.

With respect to the possible competitive interactions between mosasauroids, brachauchenines, and polycotylids, three hypotheses were considered:

(1) First mosasauroids diversified following competition with plesiosaurs.
(2) At least some mosasauroids competed with contemporary plesiosaurs or seized the opportunity and occupied their niches when they were in demise or became extinct.
(3) The fates of plesiosaurs and mosasauroids were independent of each other (no suggested competitive interactions between mosasauroids and plesiosaurs).

Having the results of our Bayesian analyses in mind, we have focused on several traits related to niche occupation. Specifically, we have compared the body size and swimming abilities of mosasauroids and plesiosaurs, discussed the thermoregulation and metabolic rates in these groups, and considered their reproductive strategies and early life history. Available evidence shows that the earliest mosasauroids differed from plesiosaurs in all these traits. Earliest Late Cretaceous (Cenomanian–Turonian) plesiosaurs were large generalists, excellent swimmers, giving birth to a single large progeny, and with metabolic rates higher than those of contemporary mosasauroids. In turn, the first mosasauroids were small, semi-aquatic animals that gave birth to multiple offspring. These marked differences, however, began to blur in the Turonian. Around the time the last brachauchenines died out (middle Turonian; though see Madzia (2016) for possible younger occurrences) or immediately after the demise of the clade, mosasauroids experienced high evolutionary rates; they evolved first larger-sized taxa and apparently also first good swimmers (e.g., Bell, Barnes & Polcyn, 2013; Polcyn et al., 2014). No evidence suggests substantial changes in the metabolic rates or reproductive strategies of larger-sized mosasauroids. In fact, preliminary reports seem to indicate that even larger mosasauroid taxa gave birth to multiple progenies (e.g., Bell & Sheldon, 2004). Still, we refrain from drawing any far-going conclusions based on the available data as the evolutionary meaning of the discussed differences is unknown.

Considering the record of direct mosasauroid-plesiosaur interactions, the fates of plesiosaurs and mosasauroids were probably not directly independent of each other. At least two mosasauroid groups containing large taxa, the tylosaurines and advanced mosasaurines, clearly interacted with polycotylids (Everhart, 2004; Einarsson et al., 2010). However, the assertion that the extinction of the last brachauchenines had been accelerated by the diversification of the tylosaurine russellosaurinans or that tylosaurines competed for and eventually ‘usurped’ the niche previously occupied by pliosaurid plesiosaurs would be speculative. The early radiation of polycotylids during the Early/Late Cretaceous transitional interval apparently produced a burst of disparity in the clade (Fischer et al., 2018), which could have had an impact on brachauchenines as well. Fischer et al. (2018) noted that the increased disparity was not an aftermath of the extinction of ichthyosaurs and pliosaurids, and that the vanishing of the high disparity in polycotylids during and after the Turonian is consistent with a model of ‘early experimentation/late constraint’. Considering the suspiciously thalassophonean-like body plans in the mid-Cretaceous polycotylid plesiosaurs, such as *Edgarosaurus muddi*, *Mauriciosaurus fernandezi*, and *Plesiopleurodon wellesi*, that co-occurred with the last pliosaurids (at a time pliosaurids show low evolutionary rates), it is possible, nevertheless, that competition with polycotylids could have contributed to the extinction of Pliosauridae.

The potential role of mosasauroids in re-shaping the early Late Cretaceous marine environments is unclear. However, we speculate that the demise of brachauchenines and decrease in both, phylogenetic diversity and disparity in polycotylids around the time mosasauroids experienced high evolutionary rates, might have resulted in that some mosasauroids, such as tylosaurines, seized the opportunity and inhabited the niche previously occupied by robust-toothed short-necked plesiosaurs. However, further clade- and trait-specific studies are necessary in order to elucidate the patterns of niche occupation in Late Cretaceous seas.

##  Supplemental Information

10.7717/peerj.8941/supp-1 Supplemental Information 1 Mosasauroid and plesiosaur FADs and LADs and the geographic distribution

10.7717/peerj.8941/supp-2 Supplemental Information 2 BEAST file for the Bayesian analysis of Mosasauroidea

10.7717/peerj.8941/supp-3 Supplemental Information 3 NEXUS file with the dataset of Mosasauroidea

10.7717/peerj.8941/supp-4 Supplemental Information 4 Character list for the phylogenetic analyses of Mosasauroidea

10.7717/peerj.8941/supp-5 Supplemental Information 5 BEAST file for the Bayesian analysis of Plesiosauria

10.7717/peerj.8941/supp-6 Supplemental Information 6 NEXUS file with the dataset of Plesiosauria

10.7717/peerj.8941/supp-7 Supplemental Information 7 Character list for the phylogenetic analyses of Plesiosauria

10.7717/peerj.8941/supp-8 Supplemental Information 8 Maximum Clade Credibility Tree (MCCT) of Plesiosauria Full phylogenetic tree.

10.7717/peerj.8941/supp-9 Supplemental Information 9 Log files from the Bayesian analyses of Mosasauroidea and Plesiosauria

## Institutional abbreviations

CAMSM: Sedgwick Museum of Earth Sciences, University of Cambridge, Cambridge, UK
IRSNB: Royal Belgian Institute of Natural Sciences, Brussels, Belgium
ROM: Royal Ontario Museum, Toronto, Canada
TMM: Texas Memorial Museum, University of Texas at Austin, Austin, Texas, USA

## Additional Information and Declarations

### Data Availability

The following information was supplied regarding data availability: The data matrices used for Bayesian analyses and resulting log files are available in the Supplemental Files.
