# Supplementary material for: Estimating the evolutionary rates in mosasauroids and plesiosaurs: discussion of niche occupation in Late Cretaceous seas
Source: PeerJ. 2020 Apr 13;8:e8941. doi: 10.7717/peerj.8941 (PMC7164395; doi:10.7717/peerj.8941)
Supplement: Supplemental Information 4 [file peerj-08-8941-s004.docx]

**Character list for the phylogenetic analysis of Mosasauroidea.**

The character list was taken from Simões et al. (2017) and formatted.

See Simões et al. (2017) for detailed comments on each of the characters.

**1. Premaxilla predental rostrum I:** total lack of a bony rostrum (0); or presence of any predental rostrum (1).

**2. Premaxilla predental rostrum II:** rostrum very short and obtuse (0); or distinctly protruding (1); or very large and inflated (2).

**3. Premaxilla shape:** bone broadly arcuate anteriorly (0); or relatively narrowly arcuate or acute anteriorly (1).

**4. Premaxilla internarial bar** **width:** narrow, distinctly less than half of the maximum width of the rostrum in dorsal view (0); or wide, being barely narrower than the rostrum (1).

**5. Premaxilla internarial bar base shape:** triangular (0); or rectangular (1).

**6. Premaxilla internarial bar dorsal keel:** absent (0); or present (1).

**7. Premaxilla internarial bar venter:** with entrance for the fifth cranial nerve close to rostrum (0); or far removed from rostrum (1).

**8. Frontal shape in front of the orbits:** sides sinusoidal (0); or bone nearly triangular and sides relatively straight (1).

**9. Frontal width:** element broad and short (0); intermediate dimensions (1); or long and narrow (2).

**10. Frontal narial emargination:** frontal not invaded by posterior end of nares (0); or distinct embayment present (1).

**11. Frontal midline dorsal keel:** absent (0); or low, fairly inconspicuous (1); or high, thin, and well-developed (2).

**12. Frontal ala shape:** sharply acuminate (0); or more broadly pointed or rounded (1).

**13. Frontal olfactory canal embrasure:** canal not embraced ventrally by descending processes (0); or canal almost or completely enclosed below (1).

**14. Frontal posteroventral midline:** tabular boss immediately anterior to the frontal-parietal suture absent (0); or present (1).

**15. Frontal-parietal suture:** apposing surfaces with low interlocking ridges (0); or with overlapping flanges (1).

**16. Frontal-parietal suture overlap orientation:** suture with oblique median frontal and parietal ridges contributing to overlap (0); or with all three ridges almost horizontal (1).

**17. Frontal invasion of parietal I:** lateral sutural flange of frontal posteriorly extended (0); or median frontal sutural flange posteriorly extended (1); or both extended (2); or suture straight (3).

**18. Frontal medial invasion of parietal II:** if present, posteriorly extended median sutural flange short (0); or long (1).

**19. Parietal length:** dorsal surface relatively short with epaxial musculature insertion posterior, between suspensorial rami only (0); or dorsal surface elongate, with epaxial musculature insertion dorsal as well as posterior (1).

**20. Parietal table shape:** generally rectangular to trapezoidal, with sides converging, but not meeting (0); or triangular, with sides contacting in front of suspensorial rami (1); or triangular table with posterior portion forming parasagittal crest or ridge (2).

**21. Parietal foramen size:** relatively small (0); or large (1).

**22. Parietal foramen position I:** foramen generally nearer to center of parietal table, well away from frontal-parietal suture (0); or close to or barely touching suture (1); or huge foramen straddling suture and deeply invading frontal (2).

**23. Parietal foramen ventral opening:** opening is level with main ventral surface (0); or opening surrounded by a rounded, elongate ridge (1).

**24. Parietal posterior shelf:** presence of a distinct horizontal shelf projecting posteriorly from between the suspensorial rami (0); or shelf absent (1).

**25. Parietal suspensorial ramus compression:** greatest width vertical or oblique (0); or greatest width horizontal (1).

**26. Parietal union with supratemoral:** suspensorial ramus from parietal overlaps supratemporal without interdigitation (0); or forked distal ramus sandwiches proximal end of supratemporal (1).

**27. Prefrontal supraorbital process:** process absent, or present as a very small rounded knob (0); or a distinct, to large, triangular, or rounded overhanging wing (1).

**28. Prefrontal contact with postorbitofrontal:** no contact at edge of frontal (0); of elements in contact there (1).

**29. Prefrontal-postorbitofrontal overlap:** prefrontal overlapped ventrally by postorbitofrontal (0); or prefrontal overlapped laterally (1).

**30. Postorbitofrontal shape:** narrow (0); or wide (1).

**31. Postorbitofrontal transverse dorsal ridge:** absent (0); or present (1).

**32. Maxilla tooth number:** 20–24 (0); or 17–19 (1); or 15–16 (2); 12–14 (3).

**33. Maxillo-premaxillary suture posterior terminus:** suture ends above a point that is anterior to or level with the midline of the fourth maxillary tooth (0); or between the fourth and ninth teeth (1); or level with or posterior to the ninth tooth (2).

**34. Maxilla posterodorsal process:** recurved wing of maxilla dorsolaterally overlaps a portion of the anterior end of the prefrontal (0); or process absent (1).

**35. Maxilla posterodorsal extent:** recurved wing of maxilla prevents emargination of prefrontal on dorsolateral edge of external naris (0); or does not (1).

**36. Jugal posteroventral angle:** angle very obtuse or curvilinear (0); or slightly obtuse, near 120^o^ (1); or 90^o^ (2).

**37. Jugal posteroventral process:** absent (0); or present (1).

**38. Ectopterygoid contact with maxilla:** present (0); or absent (1).

**39. Pterygoid tooth row elevation:** teeth arise from robust, transversely flattened, main shaft of pterygoid (0); or teeth arise from thin pronounced vertical ridge (1).

**40. Pterygoid tooth size:** anterior teeth significantly smaller than marginal teeth (0); or anterior teeth large, approaching size of marginal teeth (1).

**41. Quadrate suprastapedial process length:** process short, ends at a level well above midheight (0); or of moderate length, ending very near midheight (1); or long, distinctly below midheight (2); suprastapedial process absent (3).

**42. Quadrate suprastapedial process constriction:** distinct dorsal constriction (0); or virtually no dorsal constriction (1).

**43. Quadrate suprastapedial ridge:** if present, ridge on ventromedial edge of suprastapedial process indistinct, straight and/or narrow (0); or ridge wide, broadly rounded, and curving downward, especially above stapedial pit (1).

**44. Quadrate suprastapedial process fusion:** no fusion present (0); or process fused to, or in extensive contact with, elaborated process from below (1).

**45. Quadrate stapedial pit shape:** pit broadly oval to almost circular (0); or relatively narrowly oval (1); or extremely elongate with a constricted middle (2).

**46. Quadrate posteroventral ascending tympanic rim condition:** ascending ridge small or absent (0); or a high, elongate triangular crest (1); or a crest extremely produced laterally (2).

**47. Quadrate ala thickness:** ala thin (0); or thick (1).

**48. Quadrate conch:** ala and main shaft encompassing a deeply bowled area (0); or alar concavity shallow (1).

**49. Basisphenoid pterygoid process shape:** process relatively narrow with articular surface facing mostly anterolaterally (0); or somewhat thinner, more fan- shaped with a posterior extension of the articular surface causing a more lateral orientation (1).

**50. Quadrate ala groove:** absent (0); or long, distinct, and deep groove present in anterolateral edge of ala (1); or groove along dorsal margin of quadrate ala (2).

**51. Quadrate median ridge:** single thin, high ridge, dorsal to ventral (0); or ridge low and rounded with divergent ventral ridges (1).

**52 Quadrate anterior ventral condyle modification:** no upward deflection of anterior edge of condyle (0); or distinct deflection present (1).

**53. Quadrate ventral condyle:** condyle saddle-shaped, concave in anteroposterior view (0); or gently domed, convex in any view (1).

**54. Basioccipital tubera size:** short (0); or long (1).

**55. Basioccipital tubera shape:** tubera not anteroposteriorly elongate (0); or anteroposteriorly elongate with rugose ventrolateral surfaces (1).

**56. Basioccipital canal:** absent (0); or present as a pair separated by a median septum (1); or present as a single bilobate canal (2).

**57. Dentary tooth number:** 20–24 (0); 17–19 (1); 15–16 (2); 14 (3); 13 (4); 12 (5).

**58. Dentary anterior projection:** projection of bone anterior to first tooth present (0); or absent (1).

**59. Dentary anterior projection length:** short (0); or long (1).

**60. Dentary medial parapet:** parapet positioned at base of tooth roots (0); or elevated and strap-like, enclosing about half of height of tooth attachment in shallow channel (1), or strap equal in height to lateral wall of bone (2).

**61. Splenial-angular articulation shape:** splenial articulation in posterior view almost circular (0); or laterally compressed (1).

**62. Splenial-angular articular surface:** essentially smooth concavoconvex surfaces (0); or distinct horizontal tongues and grooves present (1).

**63. Coronoid shape:** coronoid with slight dorsal curvature, posterior wing not widely fan-shaped (0); or very concave above, posterior wing greatly expanded (1).

**64. Coronoid posteromedial process:** small but present (0); or absent (1).

**65. Coronoid medial wing:** does not reach angular (0); or contacts angular (1).

**66. Coronoid posterior wing:** without medial crescentic pit (0); or with distinct excavation (1).

**67. Surangular coronoid buttress:** low, thick, about parallel to lower edge of mandible (0); or high, thin, rapidly rising anteriorly (1).

**68. Surangular-articular suture position:** behind the condyle in lateral view (0); or at middle of glenoid on lateral edge (1); anterior to condyle (2).

**69. Surangular-articular lateral suture trace:** suture descends and angles or curves anteriorly (0); or is virtually straight throughout its length (1).

**70. Articular retroarticular process inflection:** moderate inflection, less than 60^o^ (0); or extreme inflection, almost 90^o^ (1).

**71. Articular retroarticular process innervation foramina:** no large foramina on lateral face of retroarticular process (0); or one to three large foramina present (1).

**72. Tooth surface I:** teeth finely striate medially (0); or not medially striate (1).

**73. Tooth surface II:** teeth not coarsely textured (0); or very coarsely ornamented with bumps and ridges (1).

**74. Tooth facets:** absent (0); or present (1).

**75. Tooth fluting:** absent (0); or present (1).

**76. Tooth inflation:** crowns of posterior marginal teeth conical, tapering throughout (0); or crowns of posterior marginal teeth swollen near the tip or above the base (1).

**77. Tooth carinae I:** absent (0); or present but extremely weak (1); or strong and elevated (2).

**78. Tooth carinae serration:** absent (0); or present (1).

**79. Tooth replacement mode:** replacement teeth form in shallow excavations (0); or in subdental crypts (1).

**80. Atlas neural arch:** notch in anterior border (0); or no notch in anterior border (1).

**81. Atlas synapophysis:** extremely reduced (0); or large and elongate (1).

**82. Zygosphenes and zygantra:** absent (0); or present (1).

**83. Zygosphene and zygantra number:** present on many vertebrae (0); or present on only a few (1).

**84. Hypapophyses:** last hypapophysis occurs on or anterior to seventh vertebra (0); or on eight or posteriorly (1).

**85. Synapophysis height:** facets for rib articulations tall and narrow on posterior cervicals and anterior trunk vertebrae (0); or facets ovoid, shorter than the centrum height on those vertebrae (1).

**86. Synapophysis length:** synapophyses of middle trunk vertebrae not laterally elongate (0); or distinctly laterally elongate (1).

**87. Synapophysis ventral extension:** synapophyses extend barely or not at all below ventral margin of cervical centra (0); or some extend far below ventral margin of centrum (1).

**88. Vertebral condyle inclination:** condyles of trunk vertebrae inclined (0); or condyles vertical (1).

**89. Vertebral condyle shape I:** condyles of anterior-most trunk vertebrae extremely dorsoventrally depressed (0); or essentially equidimensional (1).

**90. Vertebral condyle shape II:** condyles of posterior trunk vertebrae not higher than wide (0); or slightly compressed (1).

**91. Vertebral synapophysis dorsal ridge:** sharp ridge absent on posterior trunk synapophyses (0); or with a sharp-edged and anteriorly precipitous ridge connecting distal synapophysis with prezygapophysis (1).

**92. Vertebral length proportions:** cervical vertebrae distinctly shorter than longest vertebrae (0); or almost equal or are the longest (1).

**93. Presacral vertebrae number I:** relatively few, 32 or less (0); or numerous, 39 or more (1).

**94. Presacral vertebrae number II:** if few, then 28 or 29 (0); 30 or 31 (1).

**95. Sacral vertebrae number:** two (0); or less than two (1).

**96. Caudal dorsal expansion:** neural spines of tail all uniformly shortened posteriorly (0); or several spines dorsally elongated behind middle of tail (1).

**97. Haemal arch length:** haemal arches about equal in length to neural arch of same vertebra (0); or length about 1.5 times greater than neural arch length (1).

**98. Haemal arch articulation:** arches articulating (0); or arches fused to centra (1).

**99. Tail curvature:** no structural downturn of tail (0); or tail with curved posterior portion (1).

**100. Body proportions:** head and trunk shorter than or about equal to tail length (0); or head and trunk longer than tail (1).

**101. Scapula/coracoid size:** both bones about equal (0); or scapula about half the size of coracoid (1).

**102. Scapula width:** no anteroposterior widening (0); or distinct fan-shaped widening (1); or extreme widening (2).

**103. Scapula dorsal convexity:** if scapula widened, dorsal margin very convex (0); or broadly convex (1).

**104. Scapula posterior emargination:** posterior border of bone gently concave (0); or deeply concave (1).

**105. Scapula-coracoid suture:** unfused scapula-coracoid contact has interdigitate suture anteriorly (0); or apposing surfaces without interdigitation (1).

**106. Coracoid neck elongation:** neck rapidly tapering from medial corners to a relatively broad base (0); or neck gradually tapering to a relatively narrow base (1); coracoid neck absent (2).

**107. Coracoid anterior emargination:** present (0); or absent (1).

**108. Humerus length:** humerus distinctly elongate, about three or more times longer than distal width (0); or greatly shortened, about 1.5 to 2 times longer than distal width (1); or length and distal width virtually equal (2); or distal width slightly greater than length (3).

**109. Humerus postglenoid process:** absent or very small (0); or distinctly enlarged (1).

**110. Humerus glenoid condyle:** if present, condyle gently domed and elongate, ovoid in proximal view (0); or condyle saddle-shaped, subtriangular in proximal view and depressed (1); or condyle highly domed or protuberant and short ovoid to almost round in proximal view (2).

**111. Humerus deltopectoral crest:** crest undivided (0); or split into two separate insertional areas (1).

**112. Humerus pectoral crest:** located anteriorly (0); or medially (1).

**113. Humerus ectepicondylar groove:** groove or foramen present on distolateral edge (0); or absent (1).

**114. Humerus ectepicondyle:** absent (0); or present as a prominence (1).

**115. Humerus entepicondyle:** absent (0); or present as a prominence (1).

**116. Radius shape:** radius not expanded anterodistally (0); or slightly expanded (1); or broadly expanded (2).

**117. Ulna contact with centrale:** broad ulnare prevents contact (0); or ulna contacts centrale (1).

**118. Radiale size:** large and broad (0); or small to absent (1).

**119. Carpal reduction:** carpals number six or more (0); or five or less (1).

**120. Pisiform:** present (0); or absent (1).

**121. Metacarpal I expansion:** spindle-shaped, elongate (0); or broadly expanded (1).

**122. Phalanx shape:** phalanges elongate, spindle-shaped (0); or blocky, hourglass-shaped (1).

**123. Ilium crest:** crest blade-like, articulates with sacral ribs (0); or elongate, cylindrical, does not articulate with sacral ribs (1).

**124. Ilium acetabular area:** arcuate ridge supertending acetabulum (0); or acetabulum set into broad, short ‘V’-shaped notch (1).

**125. Pubic tubercle condition:** tubercle an elongate protuberance located closer to the midlength of the shaft (0); or a thin semicircular crest-like blade located close to the acetabulum (1).

**126. Ischiadic tubercle size:** elongate (0); or short (1).

**127. Astragalus:** notched emargination for the crural foramen, without pedunculate fibular articulation (0); or without notch, pedunculate fibular articulation present (1).

**128. Appendicular epiphyses:** formed from ossified cartilage (0); or from thick unossified cartilage (1); or epiphyses missing or extremely thin (2).

**129. Hyperphalangy:** absent (0); or present (1).

**130. Posterior thoracic vertebra:** not markedly longer than anterior thoracic vertebrae (0); or are markedly longer (1).

**131. Ectopterygoid process of pterygoid:** distal portion of process not offset anterolaterally and/or lacking longitudinal grooves and ridges (0); distal portion of process is offset anterolaterally and bears longitudinal grooves and ridges (1).
