# Supplementary figures and images for: Estimating the evolutionary rates in mosasauroids and plesiosaurs: discussion of niche occupation in Late Cretaceous seas
Source: PeerJ. 2020 Apr 13;8:e8941. doi: 10.7717/peerj.8941 (PMC7164395; doi:10.7717/peerj.8941)

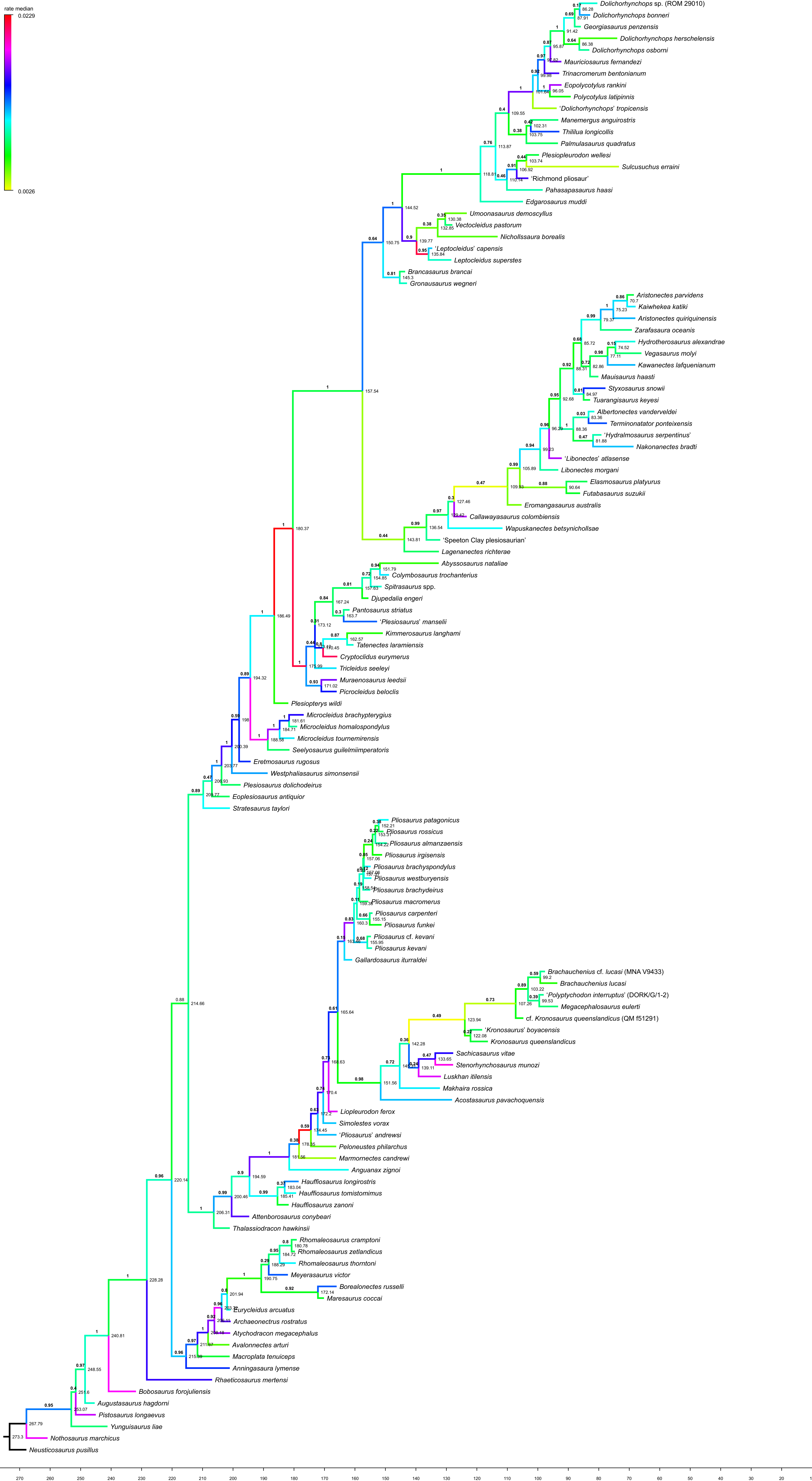

Supplement: Supplemental Information 8 — Full phylogenetic tree. [file peerj-08-8941-s008.pdf]
